# Focal adhesion kinase contributes to proliferative potential of ErbB2 mammary tumour cells but is dispensable for ErbB2 mammary tumour induction *in vivo*

**DOI:** 10.1186/bcr3131

**Published:** 2012-02-28

**Authors:** Hicham Lahlou, Virginie Sanguin-Gendreau, Margaret C Frame, William J Muller

**Affiliations:** 1 Goodman Cancer Centre, McGill University, 1160 Pine Ave. west, Montreal, QC, H3A 1A3, Canada; 2 Departments of Biochemistry and Medicine, McGill University, 3655 Promenade Sir William Osler, Montreal, QC, H3G 1Y6, Canada; 3 Edinburgh Cancer Research UK Centre, Institute of Genetics and Molecular Medicine, University of Edinburgh, Crewe Road, EH4 2XR, Edinburgh, UK

## Abstract

**Introduction:**

Activation of focal adhesion kinase (FAK) is hypothesized to play an important role in the pathogenesis of human breast cancer.

**Methods:**

To directly evaluate the role of FAK in mammary tumour progression, we have used a conditional FAK mouse model and mouse mammary tumour virus (MMTV)-driven Cre recombinase strain to inactivate FAK in the mammary epithelium of a transgenic mouse model of ErbB2 breast cancer.

**Results:**

Although mammary epithelial disruption of FAK in this model resulted in both a delay in onset and a decrease in the number of neoplastic lesions, mammary tumours occurred in 100% of virgin female mice. All of the tumours and derived metastases that developed were proficient for FAK due to the absence of Cre recombinase expression. The hyperplastic epithelia where Cre-mediated recombination of FAK could be detected exhibited a profound proliferative defect. Consistent with these observations, disruption of FAK in established tumour cells resulted in reduced tumour growth that was associated with impaired proliferation. To avoid the selection for FAK-proficient ErbB2 tumour epithelia through escape of Cre-mediated recombination, we next intercrossed the FAK conditional mice with a separate MMTV-driven ErbB2 strain that co-expressed ErbB2 and Cre recombinase on the same transcriptional unit.

**Conclusions:**

While a delay in tumour induction was noted, FAK-deficient tumours arose in 100% of female animals indicating that FAK is dispensable for ErbB2 tumour initiation. In addition, the FAK-null ErbB2 tumours retained their metastatic potential. We further demonstrated that the FAK-related Pyk2 kinase is still expressed in these tumours and is associated with its downstream regulator p130Cas. These observations indicate that Pyk2 can functionally substitute for FAK in ErbB2 mammary tumour progression.

## Introduction

Elevated expression of FAK has been associated with highly invasive human breast cancers [1,2]. In particular, several groups have reported a correlation between FAK and human epithelial growth factor receptor (ErbB2, Neu) overexpression in ErbB2-positive human breast cancer [3-5]. Activation of FAK has also been observed in human breast cancer cell lines expressing elevated levels of ErbB2 [6,7]. Moreover, recent studies have indicated that FAK and the related kinase Pyk2 are expressed in ErbB2-positive breast cancer and contribute to the proliferative and invasive potential of breast cancer cell lines [8,9].

Direct evidence for the *in vivo *importance of FAK in tumourigenesis derives from several recent studies in which components of the integrin signaling pathway were selectively ablated in the germline of mice. For example, mice heterozygous for a FAK null allele exhibit a dramatic delay in tumour induction in a chemically-induced skin carcinogenesis model [10]. Because germline deletion of FAK results in embryonic lethality [10,11], it is difficult to assess whether complete ablation of FAK could impact on chemical skin carcinogenesis. To circumvent this limitation, the same group demonstrated that conditional ablation of FAK in the skin resulted in an absolute block in the progression of benign papilloma lesions to malignant carcinomas in this model [12]. More recently, it has been shown that prostate-specific ablation of FAK in an SV40 T antigen mouse model resulted in the inability of prostate tumours to progress to the aggressive neuroendocrine phenotype [13].

Although these studies have largely focused on tissues such as skin, there is compelling evidence suggesting that activation of FAK is directly involved in the induction of mammary tumours *in vivo*. In the polyomavirus middle T (PyVmT) model of mammary tumour progression, mammary epithelial disruption of FAK prevented the transition of mammary hyperplastic growths into mammary adenocarcinomas [14]. This result was subsequently confirmed by a number of independent laboratories [15-17]. By contrast, another group claimed that FAK function in PyVmT tumour progression played a critical role in the initial progression of primary epithelium to the hyperplastic state [17]. The minor difference between these groups likely reflects the timing at which FAK-deficient lesions were monitored. Another suggested explanation is that the block in PyVmT tumour progression incurred by abrogation of FAK signaling was due to a deficit in the tumour-initiating cell population [15]. Taken together, these observations confirm that FAK plays a critical role in converting PyVmT mammary epithelial hyperplasias into the malignant phenotype.

Given the dramatic impact of FAK deletion on PyVmT tumour induction, we evaluated whether deletion of FAK in an activated ErbB2 mouse model resulted in a comparable phenotype. To accomplish this, we first intercrossed the mouse mammary tumour virus (MMTV)-activated ErbB2 strain (NDL2-5) to separate strains of mice bearing the MMTV-Cre and conditional FAK alleles and monitored virgin female cohorts for the development of mammary tumours. Although a delay in mammary tumour onset was observed, all animals developed mammary tumours that were highly metastatic. Genetic and biochemical analyses revealed that the majority of these tumours had escaped Cre-mediated recombination of the conditional FAK alleles and were proficient for FAK expression, indicating a strong selection for retention of FAK signaling. Cre-mediated deletion of FAK in established ErbB2 mammary tumour cells resulted in a profound delay in tumour growth that was further associated with a proliferative defect. Finally, using a transgenic mouse model that co-expressed ErbB2 and Cre recombinase, we demonstrated that while mammary epithelial ablation of FAK delayed tumour onset and reduced the number of neoplastic lesions, animals developed mammary tumours with 100% penetrance. Collectively these observations argue that while FAK contributes to ErbB2 tumour cell proliferation, it is ultimately dispensable for ErbB2 mammary tumour initiation and progression.

## Materials and methods

### Transgenic mice

MMTV-Cre and GTRosa26 mice were described previously [18,19]. MMTV-NDL2-5, MMTV-NIC and FAK^flox ^mice were generated and characterized as described [12,20,21]. FAK^flox ^mice were interbred with MMTV-NDL2-5, MMTV-Cre and MMTV-NIC mice and routine genotyping was performed by PCR as described [14]. Experimental and control virgin female mice were monitored for mammary tumour formation by twice-weekly palpation. All experiments involving mice were conducted in accordance with McGill University Facility Animal Care Committee guidelines which approves the use of animals (mice) for research.

### Tissue preparation, histology and β-galactosidase assay

The No. 4 inguinal mammary glands were used for wholemount analyses and the No. 3 thoracic glands for histological analyses. Mammary gland wholemounts were prepared as previously described [14]. For *in situ *β-galactosidase assays, lungs were processed as described previously [22]. For histological analyses, mammary glands, tumour samples and lung lobes were fixed in 10% neutral buffered formalin (Leica Microsystems Inc., Concord, ON, Canada) and transferred to 70% ethanol the next day. Samples were then paraffin-embedded, sectioned at 5 μm and H&E-stained. Quantitative image analysis of the mammary gland wholemounts and H&E-stained sections was performed using the Aperio ImageScope software (Vista, CA, USA). For lung lobe examinations, five step sections were performed at 50 μm intervals on lungs harvested from mice bearing tumours for 8 weeks. Pulmonary metastases were identified by microscopic analyses of H&E- or X-gal-stained sections.

### Immunostaining of tissue sections

For immunohistochemistry (IHC) and immunohistofluorescence (IHF), tissue sections were deparaffinized in xylene, endogenous peroxidase activity was blocked with 3% hydrogen peroxide in methanol, and antigen retrieval was done in 10 mmol/L sodium citrate (pH 6) using a pressure cooker (Cuisinart, Woodbridge, ON, Canada). Sections were then blocked with Power Block Universal Blocking Agent (Biogenex, Fremont, CA, USA), incubated in primary antibody as described previously [14], then incubated with biotinylated (Vector Laboratories, Burlington, ON, Canada) or Alexa Fluor 488 and 555 (Invitrogen/Life technology, Grand Island, NY, USA)-conjugated secondary antibodies for IHC or IF, respectively. Primary antibodies used for immunohistology were Cre recombinase (PRB-106, Covance, Denver, PA, USA), cleaved caspase-3 (9661, Cell Signaling, New England Biolabs, Pickering, ON, Canada), Ki67 (NCL-L-Ki67-MM1, NovoCastra, Leica Microsystems Inc.), and FAK (05-537, Millipore, Billerica, MA, USA). For IHF analysis, the slides were visualized using a Zeiss LSM 510 META confocal microscope (Carl Zeiss Canada, Toronto, ON, Canada).

### Immunoblotting and immunoprecipitations of tumour tissue

Lysates were prepared from mammary tumour tissues flash frozen in nitrogen, and immunoblots were performed as described previously [20]. Antibodies used for immunobloting analysis included Sigma (Oakville, ON, Canada) β-actin (clone AC-15, A5441) and Pyk2 (P3902), as well as Millipore phosphotyrosine (clone 4G10, 05-321) and c-Src (clone GD11, 05-184). Neu (sc-284) and CK8 (sc-101459) antibodies were from Santa Cruz Biotechnology (Santa Cruz, CA, USA) and Paxillin (610051), FAK (610087) and p130Cas (610271) were from BD Biosciences (Mississauga, ON, Canada). Phospho-p130Cas (4011), AKT (9272), phospho-AKT (9271), ERK1/2 (9102), phospho-ERK1/2 (9106), phospho-c-Src (2101), and phospho-Paxillin (2541) antibodies were purchased from Cell Signaling. All membranes were incubated with horseradish peroxidase-conjugated secondary antibodies (Jackson immunoresearch Laboratories, West Grove, PA, USA) and visualized using enhanced chemiluminescence (GE Healthcare Life Sciences, Baie d'Urfé, QC, Canada). Immunoprecipitations of lysates from mammary tissues were performed as described previously [20]. Pyk2 and phospho-p130Cas were immunoprecipitated with the same antibodies used for immunoblotting.

### Primary cell culture, transfection and viral transduction

Tumours at 8 weeks post-palpation were processed using a McIlwain tissue chopper (Mickle Laboratory Engineering, Guildford, Surrey, UK), dissociated in collagenase B/dispase II (Roche, Mississauga, ON, Canada) for 2 hours at 37°C, washed with PBS + 1mM EDTA and plated in DMEM supplemented with 2% FBS and Mammary Epithelial Growth Supplement (MEGS - Invitrogen). pMSCV retroviral vector containing Cre recombinase or empty pMSCV vector were used for virus production in 293VSV cells and subsequent transductions were carried out as previously described [23]. Transduced cell lines were selected and maintained in DMEM + mammary epithelial growth supplement (MEGS) with 2μg/ml puromycin (Sigma).

### Proliferation, migration and invasion assays

CellTiter Aqueous MTS (Promega, Madison, WI, USA) proliferation assays were performed according to the manufacturer's protocols using 2,500 cells per well in 96-well optical bottom plates (Nalge Nunc International, Rochester, NY, USA). For migration and invasion assays, cells were seeded in serum-free medium in transwell chambers (Falcon, BD Biosciences) with complete medium as a chemoattractant, and for the invasion assay chambers were coated with 5% matrigel (BD Biosciences). Cells were incubated for 24 hours, formalin-fixed, and stained with crystal violet. Cells that passed through the membrane were visualized by microscopy and pixel counts were determined using ImageJ software.

### Cell spreading assay

Cells of approximately 80% confluence were trypsinized, washed in serum-free DMEM/0.5% BSA + MEGS containing 100ug/ml soybean trypsin inhibitor (STI), and re-suspended with serum-free DMEM/0.5% BSA + MEGS. Suspension cells were seeded at 5 × 10^4 ^cells per well and allowed to adhere for either 15 or 120 minutes at 37°C in 24-well plates coated with fibronectin (BD Biosciences, #354411). At various time points, wells were washed twice with PBS and adherent cells were fixed with 10% neutral buffered formalin and then stained with crystal violet (0.1%). After extensive washing to remove the free dye, the stained cells were visualized by microscopy and cell spreading was determined by counting at least 200 cells.

### Mammary fat pad injection assay

Stable cell lines (2.5 × 10^5 ^cells in PBS) were injected into the fourth inguinal mammary fat pad of athymic nude mice (NCr; Taconic, Hudson, NY, USA). Mice were monitored twice weekly for tumour formation by palpation. Tumour growth was assessed by weekly caliper measurements.

## Results

### Mammary tumours that arise in conditional FAK mice selectively retain FAK due to loss of Cre expression

**Figure 1:**
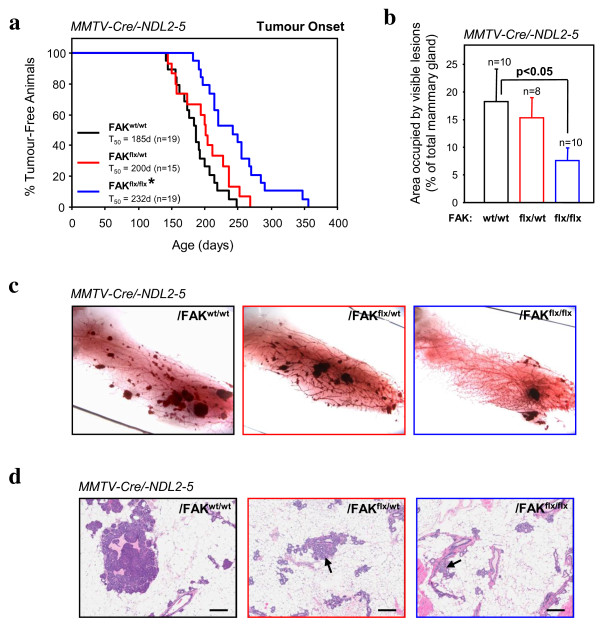
**Ablation of focal adhesion kinase (FAK) expression increases the latency of mammary tumour formation and results in fewer hyperplastic lesions in mouse mammary tumour virus (MMTV)-Cre/-NDL2-5 mice**. (**a**) Kinetics of mammary tumour onset in FAK^wt/wt^, FAK^flox/wt^, and FAK^flox/flox^/MMTV-Cre/-NDL2-5 virgin female mice. The age indicated is that at which a mammary tumour is first palpable in each transgenic strain. T_50 _denotes the age at which a tumour is palpated in 50% of the mice. *P < 0.001 vs. FAK^wt/wt ^mice, Student's *t*-test. (**b**) Quantification of the area occupied by hyperplastic lesions expressed as a percentage (± standard error of the mean, SEM) of the total mammary gland surface (**c**). Quantitative image analysis of the mammary gland wholemounts was performed using the Aperio ImageScope software. *P *< 0.05 vs. FAK^wt/wt ^mice, Student's *t-*test. (**c**) Representative fourth inguinal mammary gland wholemounts excised from late-stage tumour-bearing animals that are FAK^wt/wt ^(left), FAK^flox/wt ^(center) and FAK^flox/flox ^(right)/MMTV-Cre/-NDL2-5. Scale bar: 5 mm. (**d**) H&E-stained sections of mammary glands from 6-month-old non-tumour-bearing animals that are FAK^wt/wt ^(left), FAK^flox/wt ^(center) and FAK^flox/flox ^(right)/MMTV-Cre/-NDL2-5. Images are representative of at least five animals from each genotype and multiple fields were quantified. Scale bars: 200 μm.

To evaluate the role of FAK in ErbB2 mammary tumour progression, we interbred mice carrying conditional FAK alleles to separate transgenic strains carrying either MMTV-Cre [18] or MMTV-activated ErbB2 (NDL 2-5) [20]. We generated virgin female cohorts that carried one or both conditional FAK alleles, MMTV-Cre and MMTV-activated ErbB2 and monitored these mice for the presence of palpable mammary tumours. In contrast to the parental MMTV-activated ErbB2 strain and mice harboring one conditional FAK allele, in which mammary tumours occurred with an average onset of 200 days, animals carrying both FAK alleles developed mammary tumours with an average delayed onset of 232 days (Figure 1a). To determine whether this delay in tumour onset was reflected in a decrease in the number of pre-malignant lesions, we also performed wholemount analyses on adjacent mammary glands and scored the number of hyperplasia. The results showed that tumour-bearing mice homozygous for the floxed FAK alleles possessed fewer lesions than wild-type or heterozygous mice (Figure 1c and 1d). Quantitative analyses of mammary glands from 6-month-old non-tumour-bearing animals demonstrated that disruption of both FAK alleles resulted in a significant decrease in the area of fat pad occupied by hyperplastic lesions (Figure 1b). Taken together, these observations indicate that mammary epithelial disruption of FAK results in a dramatic reduction in ErbB2-induced tumours.

**Figure 2:**
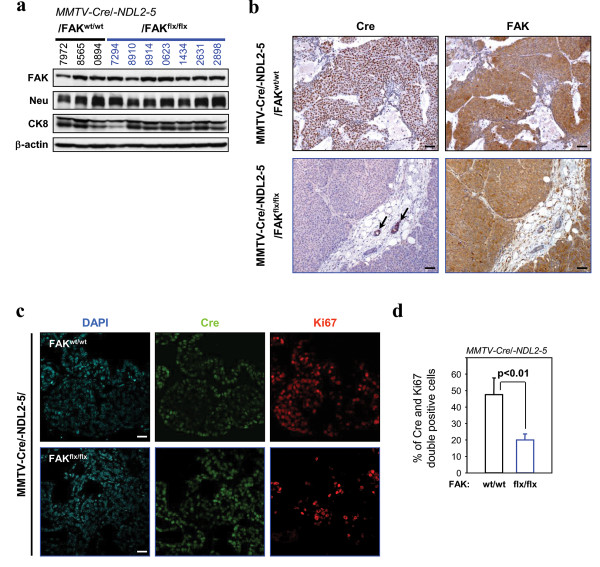
**Focal adhesion kinase (FAK)-negative mammary epithelial neoplastic cells show a reduced proliferative capacity**. (**a**) FAK and Neu expression in FAK^wt/wt ^(left) and FAK^flox/flox ^(right)/mouse mammary tumour virus (MMTV)-Cre/-NDL2-5 end-stage mammary tumours was determined by immunoblotting. Cytokeratin 8 (CK8) was used as a control for epithelial content. β-actin was used as a loading control. (**b**) Immunohistochemistry analyses of Cre (left) and FAK (right) expression on paraffin sections of end-stage mammary tumours from FAK^wt/wt ^(upper) and FAK^flox/flox ^(lower)/MMTV-Cre/-NDL2-5 animals. Data are representative of at least five animals from each genotype. FAK is co-expressed with Cre throughout the MMTV-Cre/-NDL2-5-derived solid tumour tissue. In FAK^flox/flox ^tumour sections, Cre-positive/FAK-negative cells are detectable only in small hyperplastic or non-transformed ductal structures. (Scale bars: 100 μm). (**c**) Paraffin sections of hyperplastic mammary glands from 6-month-old FAK^wt/wt ^and FAK^flox/flox^/MMTV-Cre/-NDL2-5 mice were submitted to immunohistofluorescence analyses of Cre and Ki67 expression. (Scale bars: 20 μm). (**d**) Graphical representation of the immunostaining shown in (**c**). Percentages (± standard error of the mean, SEM) were calculated after counting multiple fields from at least five animals from each genotype. *P <*0.01 vs. FAK^wt/wt ^mice, Student's *t-*test.

One possible explanation for the eventual induction of mammary tumours in FAK^flx/flx ^mice is that they are derived from a population of mammary epithelial cells that failed to express Cre recombinase. In fact the mammary tumours that eventually arose in PyVmT mice with either conditional β1 integrin alleles or conditional FAK alleles were identified as escapee populations of epithelial cells. Indeed, these cells failed to express Cre recombinase due to the stochastic nature of Cre expression in MMTV-Cre transgenic strains [14,22]. To test this possibility, we performed immunoblot analyses with FAK- and ErbB2-specific antisera on lysates from either parental MMTV-NDL2-5 tumours or tumours homozygous for the FAK conditional alleles. The results showed that tumours derived from both genotypic combinations expressed elevated levels of FAK and ErbB2 (Figure 2a). To evaluate whether retention of FAK protein was due to loss of Cre expression, we also performed immunohistochemical analyses on tumour sections from these genotypes with FAK- and Cre-specific antisera. Consistent with elevated FAK expression observed in the immunoblot analyses, tumour epithelia bearing both FAK conditional alleles failed to express Cre recombinase (Figure 2b). The inability to detect Cre recombinase in these sections was not due to the sensitivity of the antibody reagent as abundant Cre expression could be detected in adjacent normal epithelial cells (Figure 2b, see arrows). These data argue that the majority of mammary tumours that arise in this cross derive from escapee mammary epithelial cells that fail to express Cre recombinase.

Although the majority of tumours carrying both FAK null alleles were proficient for FAK expression due to the lack of Cre expression, several neoplastic glands possessed areas of epithelial cells that maintained Cre expression (Figure 2c) and therefore lost FAK expression (Figure S1a in Additional file 1). We evaluated both the proliferative and apoptotic status of these FAK-deficient hyperplastic lesions by immunostaining sections with antibodies directed against the Ki67 proliferation marker and the cleaved caspase 3 apoptotic marker. While Cre-positive mammary epithelia lacking functional FAK (Figure S1a in Additional file 1) exhibited low levels of apoptotic cell death as comparable to wild-type cells (Figure S1b and S1c in Additional file 1), they possessed a significantly reduced proliferative capacity (Figure 2c and 2d). Collectively these data indicate that FAK-ablated ErbB2 tumour cells are at a proliferative disadvantage relative to their FAK-expressing counterparts.

**Figure 3:**
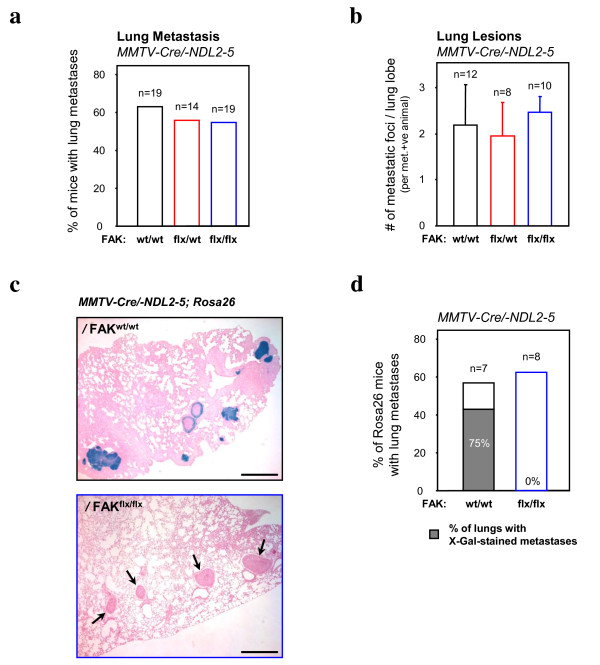
**Focal adhesion kinase (FAK)-deleted cells do not contribute to lung metastasis in the mouse mammary tumour virus (MMTV)-Cre/NDL2-5 mouse model**. (**a) **Bar graphs indicate the percentage of lung metastases for FAK^wt/wt^, FAK^flox/wt ^and FAK^flox/flox^/MMTV-Cre/-NDL2-5 mice and in (**b**) the average number of metastatic foci per lung lobe. Error bars represent the standard error of the mean (SEM). No statistically significant differences were observed (unpaired Student *t*-test). (**c**) Representative sections of X-gal-stained lung lobes from FAK^wt/wt ^(left) (n = 7) and FAK^flox/flox ^(right) (n = 8)/MMTV-Cre/NDL2-5/GTRosa26 mice bearing late-stage tumours. Arrows (lower) indicate lung lesions lacking positive X-gal staining. Scale bars: 500 μm. (**d**) Bar graphs indicate the percentage of X-gal-positive lung metastases in FAK^wt/wt ^(75% of metastases, n = 7) and FAK^flox/flox^/MMTV-Cre/-NDL2-5 mice (0% of metastases, n = 8).

Another important feature of the activated ErbB2 mammary tumour mouse model is that 60% of tumour-bearing animals will develop metastatic lesions to the lung [20]. To determine if the metastatic phase of ErbB2-induced tumour progression is affected by FAK deletion, we subjected the lungs from tumour-bearing mice to histological analyses. The tumour-bearing FAK^flx/flx ^mice developed lung metastases in the same proportion as the parental MMTV-activated ErbB2 strain (Figure 3a) with similar numbers of metastatic lesions (Figure 3b). Given that a large percentage of mammary tumours that developed in this strain were FAK-proficient due to escape from Cre-mediated recombination, it was possible that these metastatic lesions derived from escapee tumours. To test this idea, we introduced a Cre-inducible β-galactosidase reporter gene (GTRosa26 strain) [19], and counted the number of X-gal-positive lesions in lungs from either wild-type animals or mice bearing both conditional FAK alleles. Although the majority of metastatic lesions derived from MMTV-activated ErbB2 mice had undergone Cre-mediated expression of the β-galactosidase reporter, none of the metastatic lesions detected in FAK^flx/flx ^mice bearing both FAK conditional alleles were positive for X-gal, indicating that they originated from FAK-proficient escapee populations (Figure 3c and 3d). Again, these observations reinforce the concept that loss of FAK in ErbB2 tumour cells renders them at a competitive disadvantage in their capacity to colonize the metastatic site.

### Acute loss of FAK in established ErbB2 tumour cells impacts on their proliferative ability

**Figure 4:**
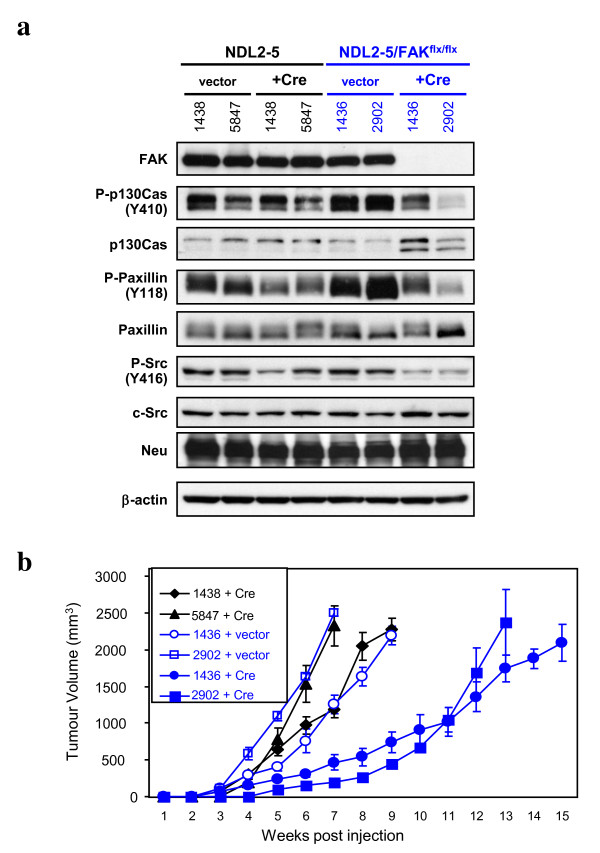
**Focal adhesion kinase (FAK) deletion in established ErbB2 tumour cells results in impaired tumour growth**. (**a**) FAK ablation in NDL2-5 cell lines decreases phosphorylation of p130Cas, Paxillin and c-Src. Cell lines derived from FAK^wt/wt ^(left side, 1438 and 5847) and FAK^flox/flox ^(right side, 1436 and 2902)/MMTV-NDL2-5 tumours are expressing an empty (vector) or Cre vector (Cre). Cells were lysed and immunoblotted for the indicated proteins. (**b**) NDL2-5/FAK^wt/wt ^cell lines expressing Cre and NDL2-5/FAK^flx/flx ^cell lines expressing empty (vector) or Cre vector (Cre) were injected into the mammary fat pads of nude mice and tumours were monitored by bi-weekly caliper measurements. The data are represented as tumour volumes (mm^3^) ± SD and plotted against time post-injection (n *= *5 mice/group).

Although the above studies indicated that FAK plays a critical role in the transition from neoplasia to full adenocarcinoma during ErbB2 tumour progression, whether FAK is required in established ErbB2 mammary tumour cells remained unclear. To test this possibility, we explanted two independent cell lines from the parental MMTV-NDL2-5 tumours and from ErbB2 induced tumours carrying both conditional FAK alleles (NDL2-5/FAK^flx/flx^) and infected them with empty vector controls or retroviral vectors expressing Cre recombinase (Figure 4). Immunoblot analyses with FAK-specific antisera of cell lysates from FAK^flx/flx ^cell lines revealed that FAK expression was lost upon infection with retroviral Cre vector, demonstrating efficient deletion of FAK (Figure 4a). In addition to FAK protein levels, we also evaluated the phosphorylation status of the FAK binding partners p130Cas, Paxillin and c-Src in these cells. Concomitant with abolition of FAK protein, we noted a significant reduction in the levels of phosphorylated c-Src, Paxillin and p130Cas (Figure 4a). Consistent with reduction in c-Src activation and p130Cas-initiated signaling, the FAK-deficient ErbB2 tumour cells exhibited a proliferative defect relative to their FAK-proficient counterparts (Figure S2a in Additional file 2). The FAK-deleted ErbB2 tumour cells also showed impairment in migration, invasion and spreading compared to control cells (Figure S2b in Additional file 2 and Figure S3 in Additional file 3). Collectively these observations indicate that loss of FAK in established ErbB2 tumour cells impacts on a combination of cell proliferation, migration, invasion and spreading.

To evaluate whether these FAK-null tumour cells were also compromised in their capacity to form mammary tumours, we transplanted them into the cleared fat pads of immunodeficient mice and monitored tumour growth. In contrast to FAK-expressing cells that formed measurable tumours by 3 to 4 weeks post-transplantation, the FAK-deficient cells formed tumours of comparable size only after 5 to 6 weeks (Figure 4b). Interestingly, immunoblot analyses with FAK-specific antibodies of tumour lysates from the transplanted FAK-deficient cells revealed the presence of FAK protein, which may reflect either stromal cell FAK levels or selective expansion of the small number of tumour cells that had not undergone Cre-mediated excision in the initial transplant (Figure S2c in Additional file 2). Taken together, these observations provide further evidence that retention of FAK signalling provides a profound proliferative advantage to ErbB2-driven tumour cells.

### FAK is dispensable for the initiation phase of ErbB2-induced mammary tumour progression

**Figure 5:**
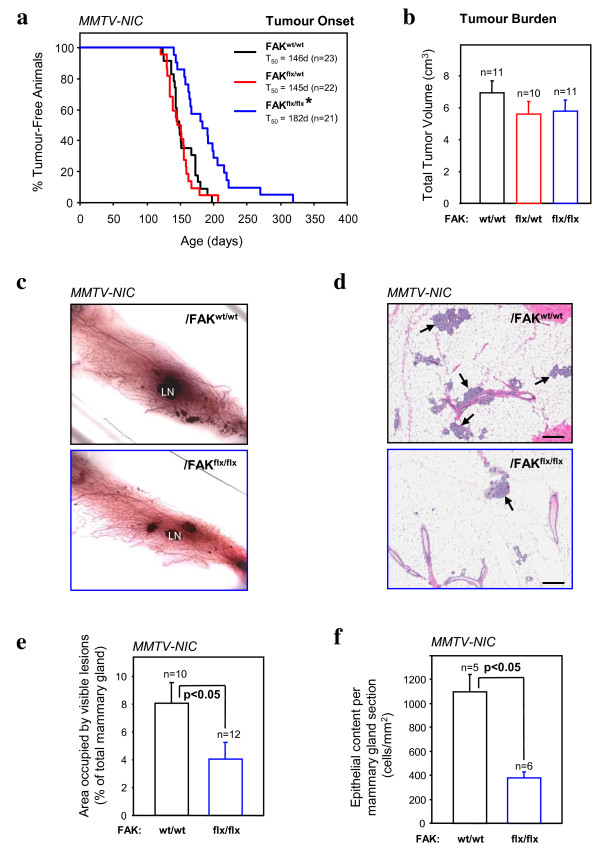
**Focal adhesion kinase (FAK) is dispensable for ErbB2-induced mammary tumour initiation and progression**. (**a**) Kinetics of mammary tumour onset in FAK^wt/wt^, FAK^flox/wt^, and FAK^flox/flox^/mouse mammary tumour virus (MMTV)-NIC virgin female mice. The age indicated is that at which a mammary tumour is first palpable in each transgenic strain. T_50 _denotes the age at which a tumour is palpated in 50% of the mice. *P < 0.001 vs. FAK^wt/wt ^mice, Student's *t*-test. (**b**) Average total tumour volume (cm^3^) in FAK^wt/wt ^(n = 11), FAK^flox/wt ^(n = 10) and FAK^flox/flox ^(n = 11)/MMTV-NIC mice. Error bars represent standard error of the mean (SEM). (**c**) Representative fourth inguinal mammary gland wholemounts excised from late-stage tumour-bearing animals from FAK^wt/wt ^(left) and FAK^flox/flox ^(right)/MMTV-NIC. LN indicates the lymph node. (**d**) H&E-stained sections from mammary glands from 4-month-old non-tumour-bearing FAK^wt/wt ^(left) and FAK^flox/flox ^(right)/MMTV-NIC animals. Images are representative of at least five animals and multiple fields were quantified. Scale bars: 200 μm. (**e**) Quantification of the area occupied by hyperplastic lesions expressed as a percentage (± standard error of the mean, SEM) of the total mammary gland surface (**c**). (**f**) Quantification of the epithelial content occupied per mammary gland section expressed as cells per surface (cells/mm^2^) (**d**). Error bars represent SEM. *P* < 0.05 vs. FAK^wt/wt ^mice, Student's *t-*test.

While the above studies provide compelling evidence that FAK contributes to ErbB2 mammary tumour progression, one limitation of this approach is that 30% of the mammary epithelial cells do not express the Cre transgene [14]. If the targeted signaling molecule is critical for tumour development, this escapee population will selectively contribute to the emerging tumour [22]. To prevent this escapee population from interfering with our analyses, we have recently developed transgenic mice that co-express both the activated form of ErbB-2 and Cre recombinase under the transcriptional control of the MMTV promoter (MMTV-NIC strain) [21]. Due to the presence of an internal ribosome entry site (IRES) between activated ErbB2 and Cre, translation of these two proteins are directly coupled from the same polycistronic mRNA. Using this unique transgenic strain, we generated cohorts of virgin female mice carrying either one or both FAK conditional alleles (Figure 5). Consistent with our previous cross with the MMTV-NDL2-5 strain, MMTV-NIC animals homozygous for both conditional FAK alleles exhibited a significant delay in tumour onset (Figure 5a) that was further associated with a deficit in the number of pre-malignant lesions in the adjacent mammary glands of tumour-bearing mice (Figure 5c and 5e) as well as in 4-month-old tumour-free mice (Figure 5d and 5f). However, once tumours developed in all of the animals, they grew to similar volumes to their parental controls (Figure 5b).

**Figure 6:**
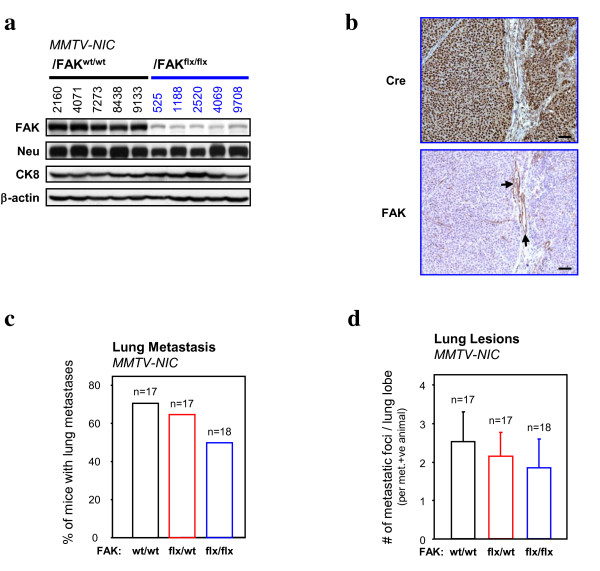
**Focal adhesion kinase FAK is dispensable for ErbB2 mammary tumour metastasis**. (**a**) FAK and Neu expression in FAK^wt/wt ^(left) and FAK^flox/flox ^(right)/mouse mammary tumour virus (MMTV)-NIC end-stage mammary tumours was determined by immunoblotting. Cytokeratin 8 (CK8) was used as a control for epithelial content. β-actin was used as a loading control. Data are representative of at least 10 animals from each genotype. (**b**) Immunohistochemistry analyses of Cre (upper) and FAK (lower) expression on paraffin sections from FAK^flox/flox^/MMTV-NIC end-stage mammary tumours. FAK expression is detectable only in the stromal compartment, whereas normal mammary ducts are Cre-positive and FAK-negative. Scale bars: 50 μm. (**c**) Bar graphs indicate the percentage of FAK^wt/wt^, FAK^flox/wt ^and FAK^flox/flox^/MMTV-NIC mice that developed lung metastases. (**d**) Bar graphs indicate the number of metastatic foci per lung lobe in metastasis-positive animals. Error bars represent standard error of the mean (SEM). No statistically significant differences were observed (Student's *t-*test).

To confirm that both conditional FAK alleles were deleted in tumour samples, we subjected tumour lysates to immunoblot analyses with FAK-specific antisera. FAK levels observed in tumours harbouring both conditional FAK alleles expressed dramatically reduced levels of FAK (Figure 6a). We confirmed that this basal level of FAK protein levels was due to the adjacent stroma by subjecting tumour sections to immunohistochemical analyses with both Cre- and FAK-specific antisera (Figure 6b). The ablation of FAK function was further accompanied by reduced total Paxillin protein and reduced (but not lost) tyrosine phosphorylation of FAK substrates such as p130Cas and Paxillin (Figure S4 in Additional file 4). However c-Src, ERK and AKT activation were not affected by FAK deletion (Figure S4 in Additional file 4).

Given that the ErbB2 tumour epithelia were completely devoid of FAK protein (Figure 6b), we next evaluated whether these FAK-deficient tumours were capable of forming metastatic lesions within the lung. Surprisingly, histological analyses of lung lobes from tumour-bearing females revealed that both the penetrance and number of metastatic lesions were not significantly impacted (Figure 6c and 6d).

One hypothesis for the lack of a defect in both tumour growth and metastasis is that the closely related Pyk2 kinase is functionally substituting for FAK. Indeed, functional compensation of FAK ablation by Pyk2 has previously been reported in mice lacking endothelial cell FAK [24]. Recently, a compensatory role for Pyk2 in the promotion of FAK-null mammary cancer stem cell tumourigenicity and metastatic activity has been shown in the context of the PyVmT oncogene [25]. To evaluate whether Pyk2 could be detected in FAK-null tumours, we measured Pyk2 levels and activity by immunoblot/immunopreciptation analyses with Pyk2 and phosphotyrosine-specific antisera (Figure S5 in Additional file 5). Although no difference in the levels of Pyk2 expression was observed in FAK-deficient tumours (Figure S5a and S5b in Additional file 5), Pyk2 is overexpressed in ErbB2 tumours when compared to adjacent mammary tissue that does not express high levels of activated Erbb2 (Figure S5c in Additional file 5). Loss of FAK overexpression in tumours bearing both conditional FAK alleles did not affect ErbB2-induced Pyk2 overexpression (Figure S5c in Additional file 5). Consistently, no significant difference in the levels of Pyk2 tyrosine phosphorylation was noted when comparing FAK-null tumours to controls (Figure S5d in Additional file 5). Interestingly, Pyk2 could still co-immunoprecipitate with p130Cas in FAK-ablated tumour lysates (Figure S5d in Additional file 5), suggesting that the remaining levels of p130Cas phosphorylation were due to the ability of Pyk2 to functionally substitute for FAK.

## Discussion

Elevated expression of FAK has been previously implicated as an important determinant in both the initiation and the progression of ErbB2-positive breast cancers [6,7]. Consistent with these observations, several *in vitro *studies with ErbB2-transformed mammary tumour cells have identified FAK and its closely related family member Pyk2 as critical signals in ErbB2-dependent proliferation [8,9,17]. Although these studies provide compelling data supporting a role for FAK in ErbB2 tumour progression, whether FAK is required *in vivo *for ErbB2 mammary tumour progression has not been addressed; this question is imperative given that the integrin/FAK signalling output from ErbB2 tumour cells is highly dependent on the microenvironment within the mammary fat pad. Here we demonstrate using two well-characterized transgenic mouse models of ErbB2 breast cancer that while the presence of functional FAK confers a proliferative advantage to ErbB2 tumour cells, metastatic ErbB2 mammary tumours can be generated in the complete absence of FAK expression.

To evaluate the role of FAK in ErbB2 mammary tumour progression, we initially interbred the MMTV-activated ErbB2 strain (NDL2-5) [20] with separate strains of mice bearing MMTV-Cre and conditional FAK alleles [12,18]. Although a delay in mammary tumour onset was observed (Figure 1), the majority of tumour epithelia maintained expression of FAK due to loss of Cre expression (Figure 2). This phenomenon persisted throughout the progression to metastasis since we could not detect any FAK-deficient lesions in the lungs of FAK^flx/flx ^tumour-bearing mice (Figure 3). One potential explanation for the presence of these escapee populations of tumour cells that have not undergone Cre-mediated recombination is that the few FAK-ablated tumour epithelia detected were at a proliferative disadvantage relative to their FAK-proficient counterparts (Figure 2). This observation is consistent with results from similar experiments in the PyVmT tumour model system where FAK was shown to influence the proliferative status of these tumour cells [14,16,17]. Likewise, mammary disruption of β1-integrin or c-Src signalling pathways (which are immediately upstream of FAK) also impairs PyVmT tumour cell proliferation [22,23]. Collectively these data argue that the integrin/c-Src/FAK signalling network is a key regulator of the proliferative capacity of both ErbB2 and PyVmT mammary tumour cells.

While these studies indicate the importance of FAK in the initiation phase of ErbB2 mammary tumour progression, acute deletion of FAK in established tumour cells also impacts on their ability to proliferate in both *in vitro *and *in vivo *settings (Figure 4) (Figure S2a in Additional file 2). In fact, from a population of tumour cells that were largely FAK-deficient (Figure 4a), the tumours that eventually arose regained FAK expression (Figure S2c in Additional file 2). The presence of FAK protein suggests that these tumour cells presumably derived from the small population of cells that had not undergone productive excision of the FAK conditional allele; alternatively, it could also represent FAK from the stromal compartment of the mammary gland which was the case in the FAK-null/MMTV-NIC tumours (Figure 6a and 6b). Again these data suggest that retention of a functional FAK gene confers a strong proliferative advantage to ErbB2 tumour cells over their FAK-deleted counterparts.

Although these data demonstrate that FAK activation is involved in the induction and maintenance of ErbB2 tumours, our studies with the MMTV-NIC model indicate that ErbB2 tumours completely lacking FAK can develop eventually. By contrast, deletion of FAK in the PyVmT model prevented tumour progression entirely [14]. One possible explanation for this difference is that the tumour-initiating cell population in the PyVmT model is absolutely reliant on FAK, whereas this population in the ErbB2 model can function independently of FAK signalling. Consistent with this hypothesis, FAK function has been reported to be important in tumour-initiating cells in the PyVmT model [15].

Examination of downstream signaling pathways revealed that loss of FAK resulted in a reduction in tyrosine phosphorylation of p130Cas (Figure 6). However, in contrast to complete loss of tyrosine phosphorylation of p130Cas in an ErB2 model with abrogation of either β1-integrin or integrin linked kinase (ILK) [26,27], FAK-deficient tumours still retained a basal level of tyrosine-phosphorylated p130Cas that was further correlated with its ability to couple with the FAK-related kinase Pyk2 (Figure S4 in Additional file 4 and Figure S5d in Additional file 5). Although it has been proposed that FAK was critical for migratory behaviour whereas Pyk2 was involved in the proliferative phase of ErbB2 tumour induction *in vitro *[8], the observation that FAK-null ErbB2 tumours can form metastatic lesions argues that at least in the *in vivo *setting, Pyk2 can functionally substitute for FAK. One of the main targets for Pyk2 is likely p130Cas, as it has been reported that p130Cas is essential for ErbB2 tumour progression [28]. While these data implicate the Pyk2/FAK/p130Cas signalling complex as an important signalling element in ErbB2 tumour progression, both ILK- and β-1 integrin-ablated ErbB2 tumours lack detectable tyrosine phosphorylated p130Cas [26,27] indicating that tyrosine phosphorylation of the p130Cas scaffold is not essential for ErbB2 tumour induction. However, tumours from both these models are poorly metastatic [26,27] suggesting that engagement of Pyk2/FAK/p130Cas signalling likely plays a more important role in the metastatic phase of ErbB2 tumour induction. Consistent with this concept, upregulation of Pyk2 has been observed in metastatic foci derived from PyVmT cancer stem cells devoid of FAK function [25].

One of the most interesting findings of these studies was the observation that acute knockout of FAK from established ErbB2 tumour cells (Figure 4) was more deleterious than early abrogation of FAK function in the MMTV-NIC model (Figure 6). A hypothesis for this difference in response between these two models is that established ErbB2 tumour cells have become addicted to the requirement for FAK function whereas MMTV-NIC-initiated tumour cells have evolved to circumvent the FAK signalling network through compensation by Pyk2. Indeed, the phenomenon of oncogene *addiction *has been observed in a number of human cancers [29].

## Conclusions

Collectively these observations suggest that targeting both Pyk2 and FAK will be required to effectively eliminate ErbB2-dependent mammary tumours. Indeed, it has been reported that small molecule inhibitors specific to FAK tyrosine kinases have both *in vitro *and *in vivo *efficacy in interfering with mammary tumour progression [30,31]. PF-562-271, a dual FAK/Pyk2 inhibitor with anti-tumour activity, has been successfully used in preclinical studies [25,32]. The future clinical application of therapeutic strategies against FAK/Pyk2 signalling has great potential for the treatment of primary human breast cancer.

## Abbreviations

BSA: bovine serum albumin; CK8: cytokeratin 8; DMEM: Dulbecco's modified Eagle's medium; ErbB2: human epithelial growth factor receptor; FAK: focal adhesion kinase; H&E: hematoxylin and eosin; IHC: immunohistochemistry; IHF: immunohistofluorescence; ILK: integrin-linked kinase; IRES: internal ribosome entry site; MEGS: mammary epithelial growth supplement; MMTV: mouse mammary tumour virus; PCR: polymerase chain reaction; PyVmT: polyomavirus middle T; STI: soybean trypsin inhibitor.

## Competing interests

The authors declare that they have no competing interests.

## Authors' contributions

HL and VSG were involved in generating all primary data in the paper and contributed to the writing of manuscript. MF provided the FAK conditional strain. WJM was involved in experimental design and contributed to the writing of manuscript. All authors read and approved the final manuscript.

## Supplementary Material

Additional file 1 **Figure showing that focal adhesion kinase (FAK)-negative mammary epithelial neoplastic cells do not exhibit an increase in apoptosis**. (**a**) Paraffin sections of hyperplastic mammary glands from 6-month-old FAKwt/wt and FAKflox/flox/mouse mammary tumour virus (MMTV)-Cre/-NDL2-5 mice were submitted to immunofluorescence analyses with Cre- and FAK-specific antibodies. Note that Cre-positive cells in lesions from FAKflox/flox/MMTV-Cre/-NDL2-5 mice are FAK-negative. (**b**) Paraffin sections of hyperplastic mammary glands from 6-month-old FAKwt/wt and FAKflox/flox/MMTV-Cre/-NDL2-5 mice were submitted to immunofluorescence analyses of Cre and cleaved caspase-3 expression. Scale bars: 20 μm. (**c**) Graphical representation of the immunostaining shown in (**b**). Percentages (± standard error of the mean) were calculated after counting multiple fields from at least five animals from each genotype. Click here for file

Additional file 2 **Figure showing that focal adhesion kinase (FAK)-deficient tumour cells exhibit proliferative, migratory and invasive defects**. (**a**) FAK ablation in NDL2-5 tumour cells decreases proliferation. MTS proliferation assays (n = 3) were performed on NDL2-5/FAKwt/wt and NDL2-5/FAKflx/flx cell lines expressing empty vector (vector) or Cre vector (Cre). Data are normalized to values at 24 hours for the control cells. Error bars represent standard error of the mean (SEM). *P *< 0.01, vs. empty vector control (unpaired Student's *t-*test). (**b**) Migration and invasion through matrigel was assayed in NDL2-5/FAKwt/wt and NDL2-5/FAKflx/flx cell lines expressing empty vector (vector) or Cre vector (Cre). Pixel count analyses of crystal violet-stained membranes were performed on five different fields for each cell line (n = 3). Error bars represent SEM. (**c**) Tumour lysates from FAK-deficient and -proficient NDL2-5 cell lines injected into the cleared fat pads of immunodeficient mice were immunoblotted with the indicated antibodies. β-actin was used as loading control. Click here for file

Additional file 3 **Figure showing that focal adhesion kinase (FAK)-deficient tumour cells exhibit a cell spreading defect**. (**a**) Quantification of cell spreading shown in (**b**) by counting the number of spread cells (marked by arrows in **b**) among at least 200 cells in NDL2-5/FAKwt/wt and NDL2-5/FAKflx/flx cell lines expressing empty vector (vector) or Cre vector (Cre). The percentage of spread cells (that is, cells that had become flattened) in five microscopic fields was determined. Cells were fixed for either 15 or 120 minutes after plating on fibronectin-coated slides and then stained with crystal violet. Error bars represent SEM. (**b**) Representative image of cell spreading after 120 minutes. Scale bars: 5 mm. Click here for file

Additional file 4 **Figure showing that focal adhesion kinase (FAK)-ablated tumours show reduced levels of tyrosine phosphorylated p130Cas and Paxillin**. (**a**) Lysates from FAKwt/wt and FAKflx/flx/MMTV-NIC end-stage mammary tumours were immunoblotted for the indicated proteins. Data are from 10 animals of each genotype. β-actin was used as loading control. (**b**) Quantification using ImageJ software of the immunoblots shown in (**a**) for total p130Cas and Paxillin, normalized to cytokeratin 8 (CK8) (control for epithelial content). *P* < 0.05 vs. FAKwt/wt mice, Student's *t*-test. (**c**) Quantification using ImageJ software of phosphorylation levels for the indicated proteins relative to total protein from the immunoblots shown in (**a**), normalized to CK8 (control for epithelial content). *P *< 0.05 vs. FAKwt/wt mice, Student's *t-*test. Click here for file

Additional file 5 **Figure showing that S5 Pyk2 is still overexpressed and associated with p130Cas in focal adhesion kinase (FAK)-deleted tumours**. (**a**) Pyk2 expression was determined by immunoblotting tumour lysates from FAKwt/wt and FAKflx/flx mouse mammary tumour virus (MMTV)-NIC tumours. β-actin was used as a loading control. Data are representative of at least 10 animals of each genotype. (**b**) Quantification using ImageJ software of the immunoblots shown in (**a**) for total Pyk2 expression, normalized to β-actin. (**c**) Pyk2 and ErbB2 expression in both adjacent and tumour epithelia was evaluated by immunoblot analyses with Pyk2- and ErbB2-specific antisera. β-actin was used as a loading control. (**d**) Upper: lysates from FAKwt/wt and FAKflx/flx/MMTV-NIC end-stage mammary tumours were immunoprecipitated with anti-Pyk2 (left) and anti-p130Cas (right) antibodies (n = 3). Immunoprecipitates were immunobloted with phosphotyrosine, Pyk2 and p130Cas antibodies. Lower: the bar graphs show Pyk2 phosphorylation relative to levels in FAKwt/wt from the immunoprecipitation with anti-Pyk2 (left) and Pyk2 interaction with p130Cas relative to levels in FAKwt/wt from the immunoprecipitation with anti-p130Cas (right). No significant differences were observed (unpaired Student *t-*test). Click here for file
