# Supplementary figures and images for: Focal adhesion kinase contributes to proliferative potential of ErbB2 mammary tumour cells but is dispensable for ErbB2 mammary tumour induction in vivo
Source: Breast Cancer Res. 2012 Feb 28;14(1):R36. doi: 10.1186/bcr3131 (PMC3496154; doi:10.1186/bcr3131)

Suppl. Figure 1

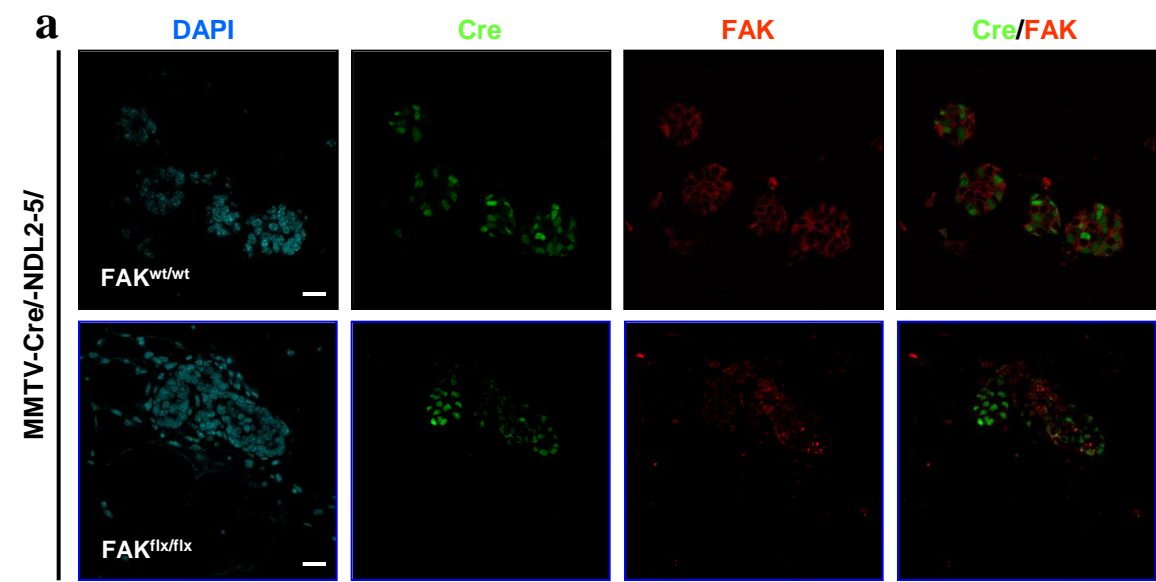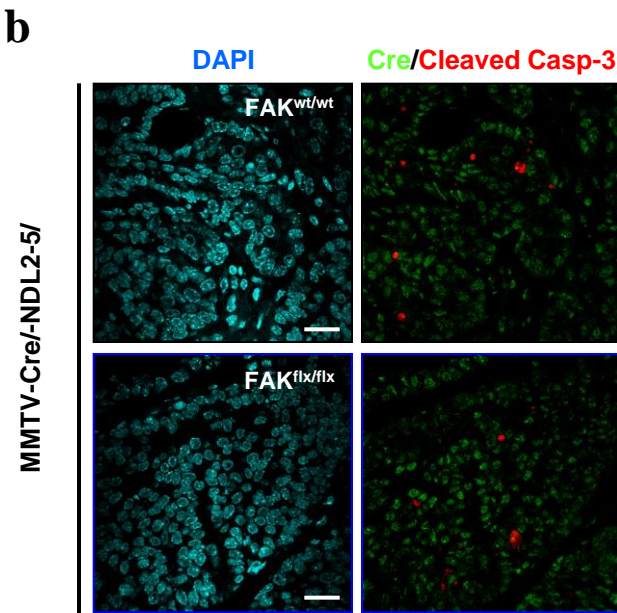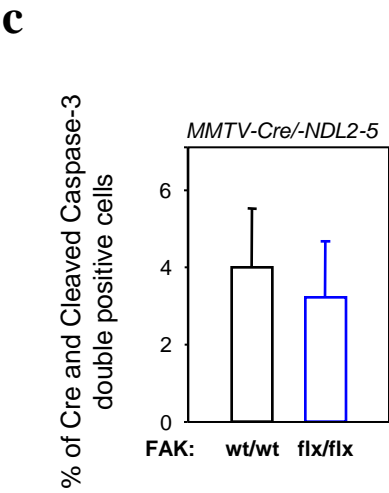

Supplement: Additional file 1 — Figure showing that focal adhesion kinase (FAK)-negative mammary epithelial neoplastic cells do not exhibit an increase in apoptosis. (a) Paraffin sections of hyperplastic mammary glands from 6-month-old FAKwt/wt and FAKflox/flox/mouse mammary tumour virus (MMTV)-Cre/-NDL2-5 mice were submitted to immunofluorescence analyses with Cre- and FAK-specific antibodies. Note that Cre-positive cells in lesions from FAKflox/flox/MMTV-Cre/-NDL2-5 mice are FAK-negative. (b) Paraffin sections of hyperplastic mammary glands from 6-month-old FAKwt/wt and FAKflox/flox/MMTV-Cre/-NDL2-5 mice were submitted to immunofluorescence analyses of Cre and cleaved caspase-3 expression. Scale bars: 20 μm. (c) Graphical representation of the immunostaining shown in (b). Percentages (± standard error of the mean) were calculated after counting multiple fields from at least five animals from each genotype. [file bcr3131-S1.PDF]

Suppl. Figure 2

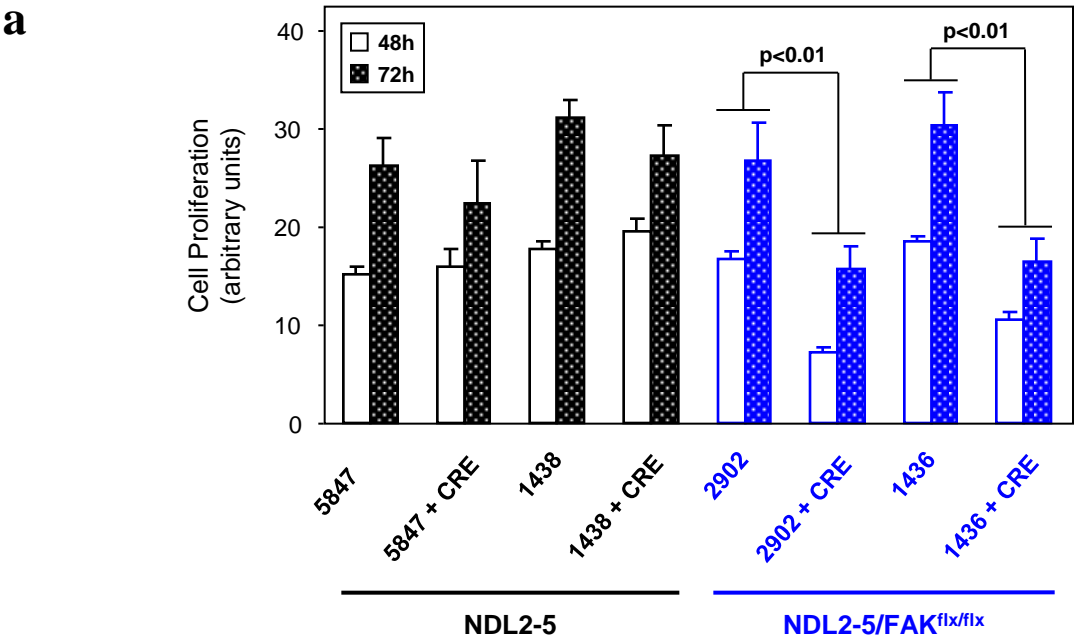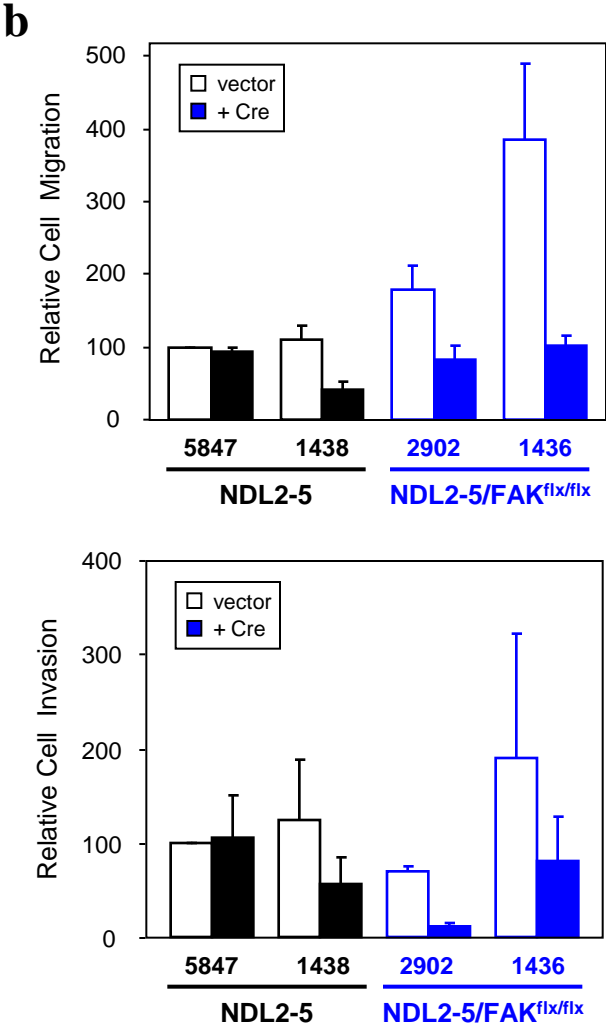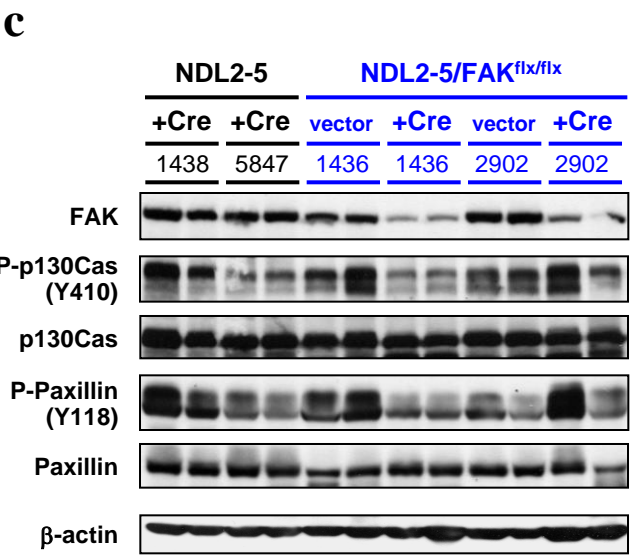

Supplement: Additional file 2 — Figure showing that focal adhesion kinase (FAK)-deficient tumour cells exhibit proliferative, migratory and invasive defects. (a) FAK ablation in NDL2-5 tumour cells decreases proliferation. MTS proliferation assays (n = 3) were performed on NDL2-5/FAKwt/wt and NDL2-5/FAKflx/flx cell lines expressing empty vector (vector) or Cre vector (Cre). Data are normalized to values at 24 hours for the control cells. Error bars represent standard error of the mean (SEM). P < 0.01, vs. empty vector control (unpaired Student's t-test). (b) Migration and invasion through matrigel was assayed in NDL2-5/FAKwt/wt and NDL2-5/FAKflx/flx cell lines expressing empty vector (vector) or Cre vector (Cre). Pixel count analyses of crystal violet-stained membranes were performed on five different fields for each cell line (n = 3). Error bars represent SEM. (c) Tumour lysates from FAK-deficient and -proficient NDL2-5 cell lines injected into the cleared fat pads of immunodeficient mice were immunoblotted with the indicated antibodies. β-actin was used as loading control. [file bcr3131-S2.PDF]

Suppl. Figure 3

a

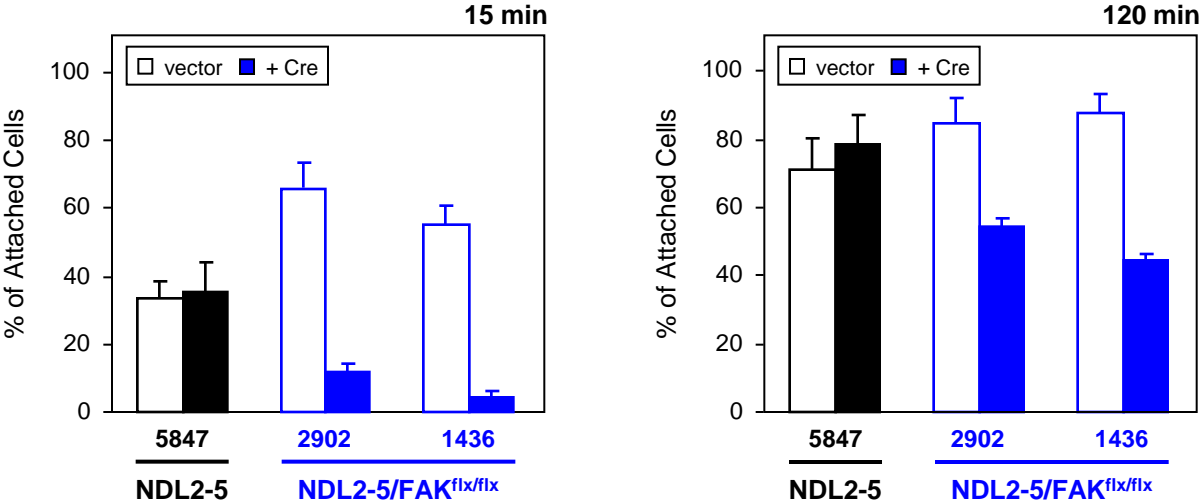

b

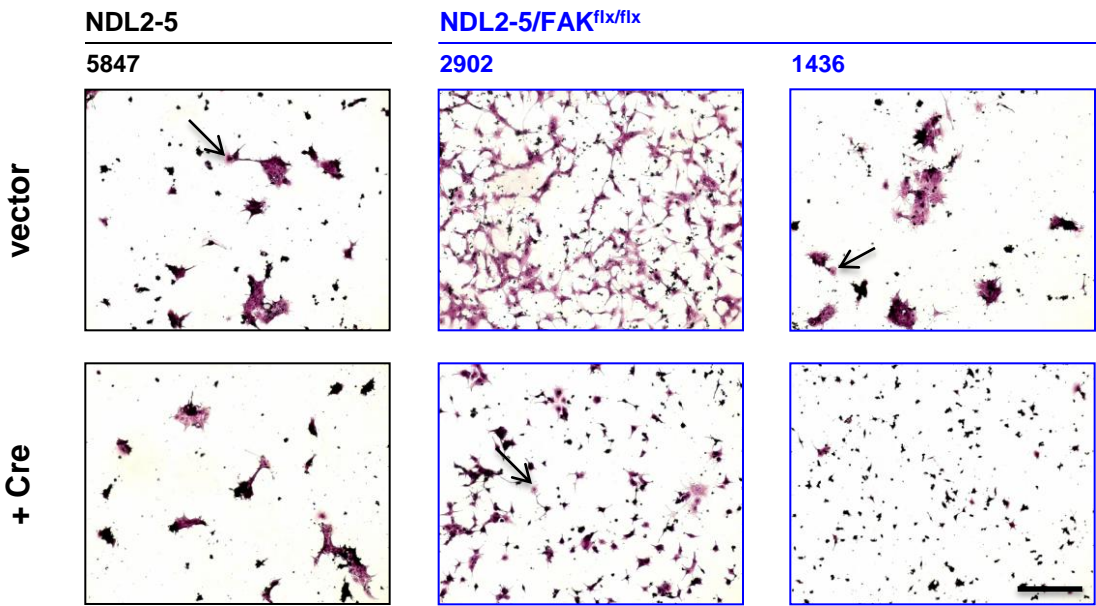

Supplement: Additional file 3 — Figure showing that focal adhesion kinase (FAK)-deficient tumour cells exhibit a cell spreading defect. (a) Quantification of cell spreading shown in (b) by counting the number of spread cells (marked by arrows in b) among at least 200 cells in NDL2-5/FAKwt/wt and NDL2-5/FAKflx/flx cell lines expressing empty vector (vector) or Cre vector (Cre). The percentage of spread cells (that is, cells that had become flattened) in five microscopic fields was determined. Cells were fixed for either 15 or 120 minutes after plating on fibronectin-coated slides and then stained with crystal violet. Error bars represent SEM. (b) Representative image of cell spreading after 120 minutes. Scale bars: 5 mm. [file bcr3131-S3.PDF]

Suppl. Figure 4

a

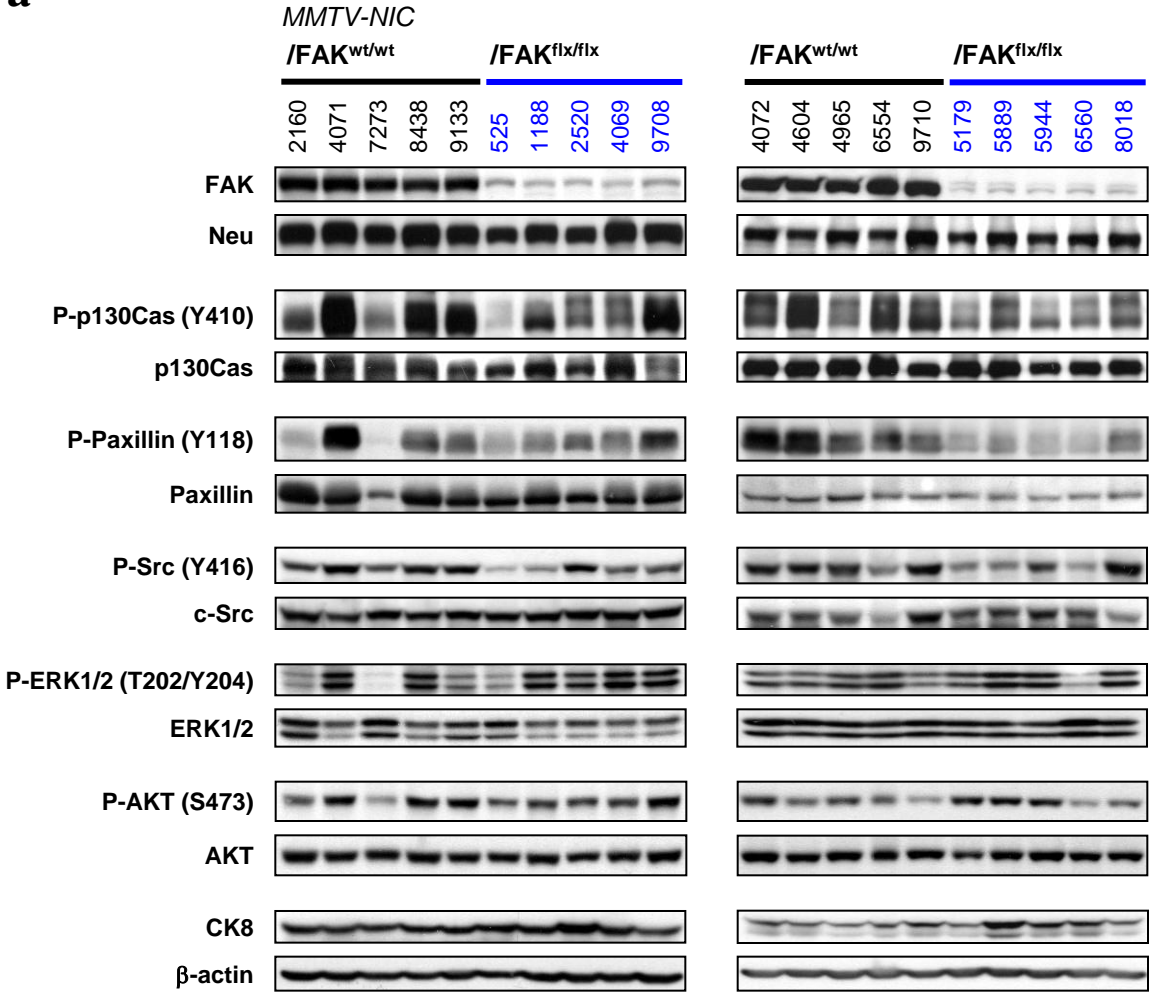

b

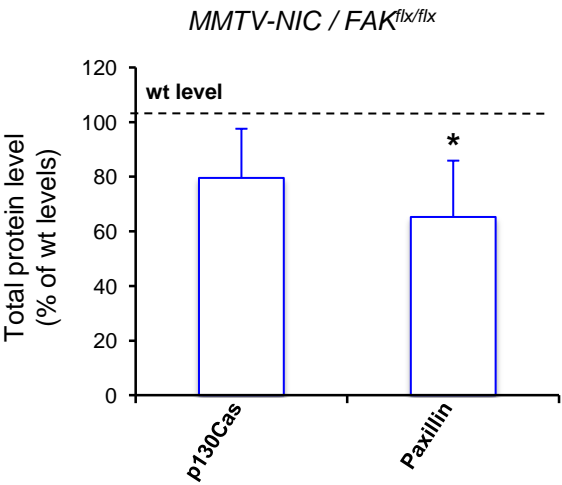

c

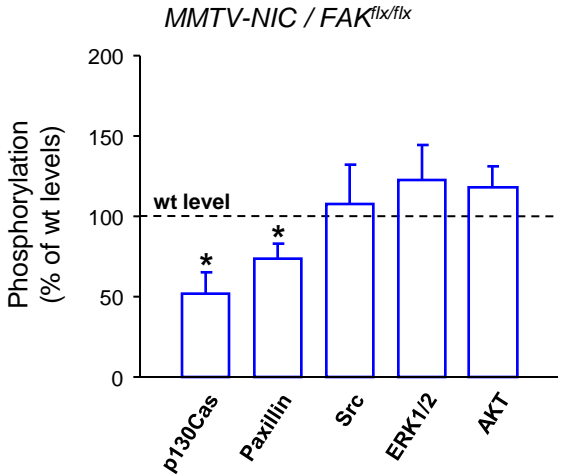

Supplement: Additional file 4 — Figure showing that focal adhesion kinase (FAK)-ablated tumours show reduced levels of tyrosine phosphorylated p130Cas and Paxillin. (a) Lysates from FAKwt/wt and FAKflx/flx/MMTV-NIC end-stage mammary tumours were immunoblotted for the indicated proteins. Data are from 10 animals of each genotype. β-actin was used as loading control. (b) Quantification using ImageJ software of the immunoblots shown in (a) for total p130Cas and Paxillin, normalized to cytokeratin 8 (CK8) (control for epithelial content). P < 0.05 vs. FAKwt/wt mice, Student's t-test. (c) Quantification using ImageJ software of phosphorylation levels for the indicated proteins relative to total protein from the immunoblots shown in (a), normalized to CK8 (control for epithelial content). P < 0.05 vs. FAKwt/wt mice, Student's t-test. [file bcr3131-S4.PDF]

Suppl. Figure 5

a

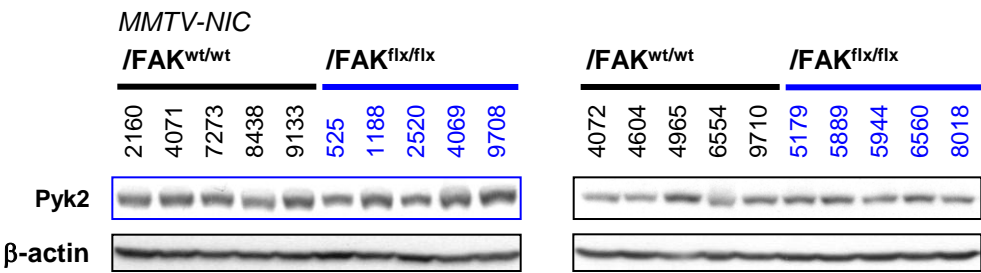

b

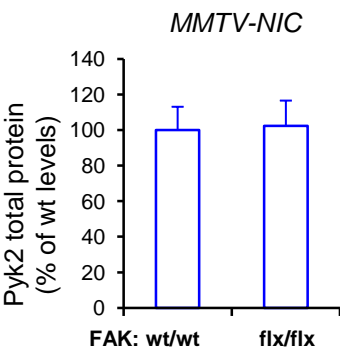

c

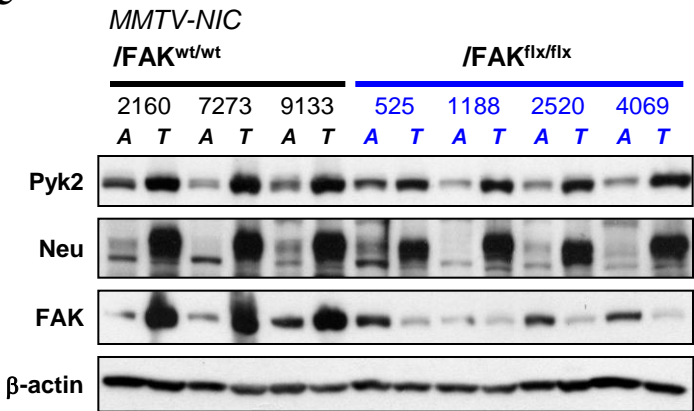

d

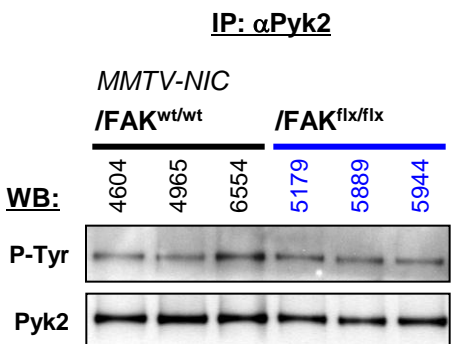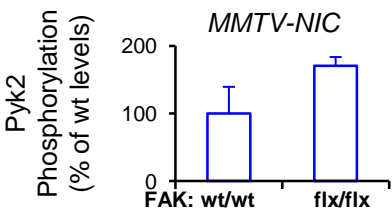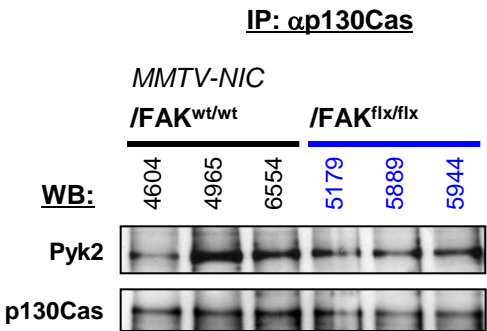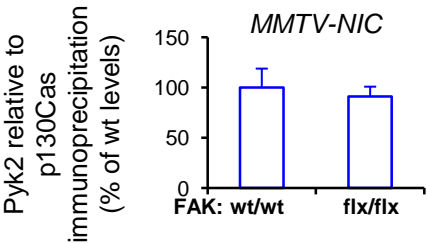

Supplement: Additional file 5 — Figure showing that S5 Pyk2 is still overexpressed and associated with p130Cas in focal adhesion kinase (FAK)-deleted tumours. (a) Pyk2 expression was determined by immunoblotting tumour lysates from FAKwt/wt and FAKflx/flx mouse mammary tumour virus (MMTV)-NIC tumours. β-actin was used as a loading control. Data are representative of at least 10 animals of each genotype. (b) Quantification using ImageJ software of the immunoblots shown in (a) for total Pyk2 expression, normalized to β-actin. (c) Pyk2 and ErbB2 expression in both adjacent and tumour epithelia was evaluated by immunoblot analyses with Pyk2- and ErbB2-specific antisera. β-actin was used as a loading control. (d) Upper: lysates from FAKwt/wt and FAKflx/flx/MMTV-NIC end-stage mammary tumours were immunoprecipitated with anti-Pyk2 (left) and anti-p130Cas (right) antibodies (n = 3). Immunoprecipitates were immunobloted with phosphotyrosine, Pyk2 and p130Cas antibodies. Lower: the bar graphs show Pyk2 phosphorylation relative to levels in FAKwt/wt from the immunoprecipitation with anti-Pyk2 (left) and Pyk2 interaction with p130Cas relative to levels in FAKwt/wt from the immunoprecipitation with anti-p130Cas (right). No significant differences were observed (unpaired Student t-test). [file bcr3131-S5.PDF]
